# Telecaregiving for Dementia: A Mapping Review of Technological and Nontechnological Interventions

**DOI:** 10.1093/geront/gnad026

**Published:** 2023-03-15

**Authors:** Jordan R Hill, Elissa E Min, Ephrem Abebe, Richard J Holden

**Affiliations:** Department of Health & Wellness Design, Indiana University School of Public Health—Bloomington, Bloomington, Indiana, USA; Indiana University Center for Aging Research, Regenstrief Institute Inc., Indianapolis, Indiana, USA; Department of Pharmacy Practice, Purdue University, West Lafayette, Indiana, USA; Department of Pharmacy Practice, Purdue University, West Lafayette, Indiana, USA; Regenstrief Center for Healthcare Engineering, Purdue University, West Lafayette, Indiana, USA; Department of Health & Wellness Design, Indiana University School of Public Health—Bloomington, Bloomington, Indiana, USA; Indiana University Center for Aging Research, Regenstrief Institute Inc., Indianapolis, Indiana, USA

**Keywords:** Aging in place, Informal caregiving, Long-distance caregiving, Remote caregiving, Technology

## Abstract

**Background and Objectives:**

Informal (or family) caregivers to older adults with Alzheimer’s disease or other related dementias (ADRD) could greatly benefit from innovative telecaregiving systems that support caregiving from a distance. The objective of this review is to better understand (a) who is involved in telecaregiving and their experiences; (b) the interventions currently available to support ADRD telecaregiving; and (c) the outcomes measured to assess the effects of ADRD telecaregiving interventions.

**Research Design and Methods:**

A mapping review was conducted by systematically searching MEDLINE, CINAHL, Embase, and PsycINFO for all works published in English from 2002 to 2022. References of included publications were searched to identify additional empirical publications for inclusion.

**Results:**

Sixty-one publications (describing 48 studies and 5 nonstudy sources) were included in the review. Currently available information on the demographics, experiences, challenges, and benefits of ADRD telecaregivers is summarized. We found that interventions to support telecaregiving could be classified into 7 categories of technological interventions and 3 categories of nontechnological interventions. Empirical studies on ADRD telecaregiving interventions investigated a variety of outcomes, the most prevalent being user experience.

**Discussion and Implications:**

We conclude that (a) the paucity of literature on telecaregiving does not allow for a comprehensive understanding of the needs and day-to-day activities of ADRD telecaregivers; (b) interventions developed to support ADRD telecaregiving may not fully meet the needs of caregivers or care recipients; and (c) there is insufficient rigorous research establishing the effects of telecaregiving interventions on key ADRD-related outcomes.

## Background and Objectives

In the United States, caregiving provided to older adults is predominantly performed by informal, unpaid caregivers such as family, friends, or neighbors. Nearly half of these caregivers care for a person living with Alzheimer’s disease or other related dementias (ADRD), at the annual rate of 16 billion hours of care, valued at approximately $271.6 billion (Alzheimer’s Association, 2022). Supporting these caregivers and their caregiving work is an urgent national priority (National Institute on Aging, 2020).

Over one in three (34%) caregivers to older adults with ADRD do not live with the care recipient and three out of five (60%) ADRD caregivers are employed, meaning they may leave the care recipient alone for an extended period (Alzheimer’s Association, 2022). Additionally, most older adults wish to stay in their homes and communities as they age (Davis, 2021), which increases the rate of care recipient isolation from formal or informal caregivers. Constant or temporary (e.g., when at work) distance between caregivers and care recipients requires what we call “telecaregiving” (cf. Bledsoe et al., [2010] and Zagaria [2012], who use the term “care from a distance”). Given the worldwide development and expansion of telehealth and telecare for professional care delivery, we argue a need to further understand telecaregiving and telecaregiving interventions as a new caregiver-centered frontier of telehealth. Our definition of telecaregiving is as follows:

- **Telecaregiving**: Informal care from a distance.
- **Telecaregiving Interventions**: Technological and nontechnological systems that support the delivery of (informal) caregiving from a distance.

The objective of the present review is to understand telecaregiving and telecaregiving interventions in the domain of ADRD caregiving. We examined published literature to answer three research questions (RQs):

*RQ1: Who is involved in ADRD telecaregiving and what are their experiences?*
*RQ2: What are the telecaregiving interventions available to support ADRD telecaregiving?*
*RQ3: What are the outcomes measured to assess the effects of ADRD telecaregiving interventions?*

To our knowledge, no contemporary review answers these questions in the domain of ADRD caregiving. Reviews have examined technological interventions, but they tend to focus on a specific kind of technology (Behera et al., 2021; Gillani & Arslan, 2021; Stavropoulos et al., 2020), are not specific to telecaregiving (Pappadà et al., 2021) or ADRD (Stavropoulos et al., 2020), and/or limit their search to a specific kind of study or outcome (Davies et al., 2013; Wang et al., 2021). A seminal but now-dated review examined dementia and older adult caregiving literature, including technological and nontechnological solutions (Bledsoe et al., 2010).

## Research Design and Methods

We performed a mapping review—as defined by Grant and Booth (2009)—to (a) map and categorize existing literature on the topics of telecaregiving and telecaregiving interventions and (b) identify gaps and future research opportunities in this area. Mapping reviews differ from other types of reviews (e.g., systematic reviews) by serving as a starting point to understand the landscape of a current research area and identify opportunities for future research, rather than critically evaluating literature to synthesize the best-quality evidence and answer a specific research question. Mapping reviews can set up either further systematic reviews or more primary research, and mapping reviews do not formally assess the quality of included publications (Grant & Booth, 2009). To more completely map the work done in this area, both peer-reviewed and nonpeer-reviewed publications, and studies of various designs (including both quantitative and qualitative designs and nonempirical literature) were included.

### Search Strategy

We systematically searched MEDLINE, CINAHL, Embase, and PsycINFO for works published in 2002–22. These keywords and phrases were searched in titles and abstracts with Boolean operators “AND,” “OR,” and “NOT”: (remote OR tele OR long-distance OR Out of town OR out-of-town) AND (caregiving OR caregiver OR caregivers OR carer OR carers) NOT (telemedicine OR rehabilitation OR telerehabilitation OR tele-rehabilitation). We did not limit our initial search results to ADRD telecaregiving as we wanted to take the opportunity to assess the available literature on telecaregiving in related fields and identify future areas of investigation. We purposely used broad search terms to pick up relevant keywords across disciplines, and therefore employed the same search strategy in all databases. The latest search was performed on December 15, 2021. The full syntax for the search performed in MEDLINE is as follows:

(((“remote”[Title/Abstract] OR “tele”[Title/Abstract] OR “long-distance”[Title/Abstract] OR “out-of-town”[Title/Abstract] OR “out-of-town”[Title/Abstract]) AND (“caregiving”[Title/Abstract] OR “caregiver”[Title/Abstract] OR “caregivers”[Title/Abstract] OR “carer”[Title/Abstract] OR “carers”[Title/Abstract])) NOT (“telemedicine”[Title/Abstract] OR “rehabilitation”[Title/Abstract] OR “telerehabilitation”[Title/Abstract] OR “tele-rehabilitation”[Title/Abstract])) AND ((english[Filter]) AND (2002:2022[pdat]))

Two authors (J. R. Hill and E. E. Min) independently screened abstracts and titles for inclusion and exclusion criteria in Covidence. Initially, included publications had to be available in English and contain content on informal telecaregiving and/or interventions to support caregiving at a distance. Reviews, tip sheets, book chapters, educational resources, case studies, and empirical studies of any design were included. J. R. Hill and E. E. Min then independently read each potentially eligible full text using the same criteria but additionally requiring that the sources provide information on ADRD telecaregiving or interventions to support ADRD telecaregiving, specifically. Publications were excluded from this review if they did not offer any information on ADRD telecaregiving or ADRD telecaregiving interventions relevant to the three RQs. The two reviewers then read each potentially eligible full text. Disagreements were resolved through discussion and consensus.

One author (J. R. Hill) systematically extracted and synthesized data from included papers using a data extraction tool pilot tested and approved by all authors. J. R. Hill also searched the Cochrane database on April 18, 2022, using the keywords to identify any overlapping systematic reviews. Another author (E. E. Min) reviewed reference lists of included publications for additional eligible empirical publications on the topic using the same inclusion criteria; 18 additional items were found through this process. Data were summarized according to the three RQs posed in this review. Upon peer review, one reviewer identified another relevant paper that was not indexed in the four databases searched, and we included it in the review.

## Results

**Figure 1.**
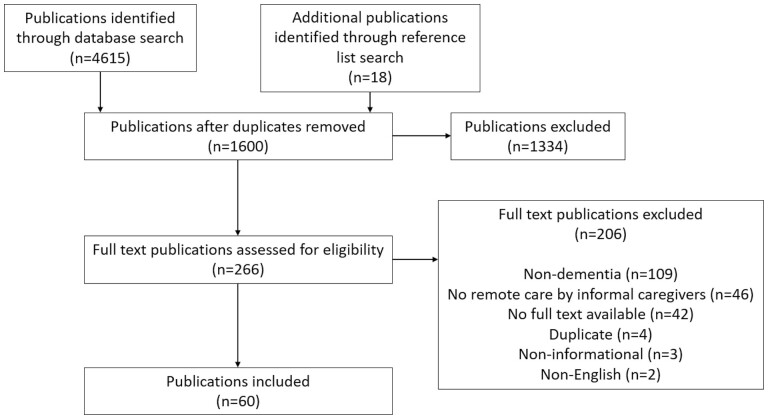
PRISMA diagram. PRISMA = Preferred Reporting Items for Systematic Reviews and Meta-Analyses.

A total of 61 publications, representing 48 separate studies and 5 non5-study sources, were found eligible for inclusion in this review (see PRISMA diagram, Figure 1). The majority of the publications found were published in the last 5 years (2016–21, 34 publications) with 2021 having the most publications on the topic in the past 20 years (15 publications). The empirical studies included in this review were performed in a wide variety of countries including the United States (11), the United Kingdom (9), Sweden (3), Canada (3), Norway (2), the Netherlands (2), Ireland (1), Germany (1), Spain (1), Romania (1), Italy (1), Taiwan (1), Japan (1), and Australia (1).

### RQ1: Demographics and Experiences of ADRD Telecaregiving

Nearly all of the information on the demographics and experiences of telecaregivers are limited to data on long-distance caregivers, who are the subset of telecaregivers who live far away from care recipients. Long-distance caregiving does not generally include, for example, caring from one’s workplace for a cohabitating care recipient, caring remotely for someone who lives nearby, or care delivered during episodes of temporary distancing such as during a hospital stay. Long-distance caregiving is variously defined, for example, based on the following:

- Travel time—for example, 1 hr (Alzheimer’s Association, 2013) or over 2 hr (Falzarano et al., 2020, 2022; Koerin & Harrigan, 2003);
- Distance—for example, living 100 km apart or more (Gunn et al., 2021);
- Access—for example, inability to be face to face due to geographical separation (Bledsoe et al., 2010) or visiting restrictions, for instance, to manage disease transmission (Behera et al., 2021; Faieta et al., 2021; Giebel et al., 2021).

Long-distance ADRD caregivers, compared to proximal caregivers, are more likely to have large families, fewer siblings, higher incomes, more education, and more extensive history of geographic mobility (Alzheimer’s Association, 2013; Bledsoe et al., 2010). Long-distance care recipients with ADRD, compared to care recipients with proximal caregivers, are more likely to have a history of geographic mobility, accrued more years of formal education, have fewer children, be younger than 80 years old, receive a pension, live alone, and be a parent of the caregiver (Alzheimer’s Association, 2013; Bledsoe et al., 2010). In the United States, long-distance caregivers and care recipients are more likely to live in the western part of the country and care recipients are more likely to live in rural communities (Alzheimer’s Association, 2013).

Similar to proximal caregivers, most long-distance caregivers of people with ADRD are middle-aged, married women (largely adult daughters) (Alzheimer’s Association, 2013; Collins et al., 2003; Koerin & Harrigan, 2003), and the majority of long-distance care recipients are female (Alzheimer’s Association, 2013). Long-distance and proximal ADRD caregivers also have similar rates of employment, about 60% employed full or part time according to older data from the Alzheimer’s Association (2013). However, female versus male long-distance caregivers of older adults in general are more likely to reduce their employment to part time or to take leaves of absence (Smith, 2006), and long-distance caregivers of people with ADRD may follow a similar trend.

Reasons compelling long-term caregiving described in the literature include:

- A more global society making it more common for children to move away (Collins et al., 2003);
- High costs of assisted living, care homes, or professional caregiving services (Behera et al., 2021);
- Lack of available care services in the vicinity of the care recipient or caregiver (Behera et al., 2021; Giebel et al., 2021).

Health and disability levels were not found to strongly influence ADRD caregiver and care recipient geographic separation (Alzheimer’s Association, 2013).

The average long-distance caregiver (of an older adult with or without ADRD) spends an estimated 5.22 years providing care (Koerin & Harrigan, 2003). They are more likely to assist with instrumental activities of daily living (IADLs; e.g., financial management, medication management, housework, shopping) than activities of daily living (ADLs; Alzheimer’s Association, 2013; Davies et al., 2013; Koerin & Harrigan, 2003; Lewis, 2008; Zagaria, 2012). However, over one third (33%–40%) also report assisting with some ADLs such as helping care recipients get in and out of bed and chairs (Alzheimer’s Association, 2013; Koerin & Harrigan, 2003); it is not clear in these sources how long-distance caregivers help with these tasks remotely (e.g., hiring professional services) or if they help with these tasks during the times they are with the care recipient. Specific to long-distance caregivers of older adults with ADRD, an average estimated 3.4 hr/week is spent arranging services for the care recipient, 4hr/week checking on the care recipient and monitoring their care, 22 hr/month helping the care recipient with IADLs, and 12 hr/month assisting with ADLs (Alzheimer’s Association, 2013).

With the development of new technologies, long-distance caregivers, in general, now perform remote monitoring or surveillance of care recipients within their homes (Davies et al., 2013), and often provide emotional support and work to keep them feeling connected to their families (Faieta et al., 2021; Lewis, 2008; Zagaria, 2012).

### Challenges and Benefits of ADRD Telecaregiving

In general, ADRD caregiving needs are compounded by distance caregiving challenges (Alzheimer’s Association, 2013; Bledsoe et al., 2010; Falzarano et al., 2020) commonly including or producing the following:

- Higher out-of-pocket expenses related to caregiving (Alzheimer’s Association, 2013; Collins et al., 2003; Koerin & Harrigan, 2003; Lewis, 2008; Smith, 2006);
- Frequent long-distance travel that can be physically and emotionally draining (Lewis, 2008);
- Difficulties finding professional services available in the care recipient’s community and difficulty in monitoring those service providers (Alzheimer’s Association, 2013; Koerin & Harrigan, 2003; Watari et al., 2006);
- Difficulty assessing the needs of care recipients, obtaining accurate information about them, and communicating with health care professionals or formal caregivers (Alzheimer’s Association, 2013; Falzarano et al., 2020, 2022; Koerin & Harrigan, 2003; Lewis, 2008; Smith, 2006);
- Lack of information on ADRD (Bledsoe et al., 2010);
- Higher levels of psychological distress (Alzheimer’s Association, 2013; Falzarano et al., 2020; Koerin & Harrigan, 2003) which are potentially even higher among African American caregivers (though this is based on a single case study, and would require further investigation; Collins et al., 2003);
- Greater disruptions in employment (Alzheimer’s Association, 2013; Collins et al., 2003; Falzarano et al., 2022; Koerin & Harrigan, 2003);
- More frequent sacrifice of vacations, hobbies, and other leisure activities due to caregiving responsibilities (Koerin & Harrigan, 2003); and
- More common family disagreements about care (Bledsoe et al., 2010; Koerin & Harrigan, 2003; Watari et al., 2006).

At the same time, the benefits of telecaregiving described in the literature include the following:

- Decreased tendency to feel overwhelmed and/or physically exhausted (Alzheimer’s Association, 2013; Bledsoe et al., 2010; Koerin & Harrigan, 2003);
- Decreased amount of unpaid labor provided compared to local caregivers (Alzheimer’s Association, 2013); and
- Higher satisfaction with formal care (Falzarano et al., 2020) and other services (Watari et al., 2006).

Additionally, Tiersen et al. (2021) identified a potential benefit of care recipients who live alone: it is easier for technological interventions to monitor care recipient activities as the technology will be less likely to falsely identify caregiver or other family member activities as those of the care recipient.

### RQ2: ADRD Telecaregiving Interventions

**Figure 2.**
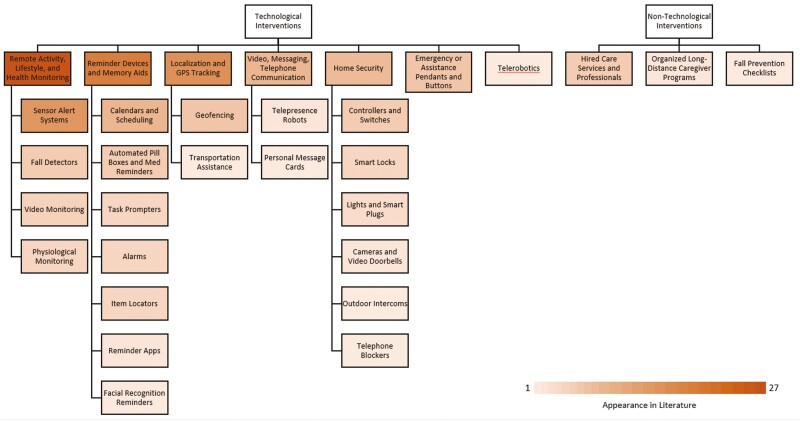
Categories of ADRD telecaregiving systems. ADRD = Alzheimer’s disease or other related dementias.

Figure 2 summarizes technological and nontechnological interventions supporting ADRD telecaregiving, organized by subtype and color coded by frequency of appearance in the literature.

#### Technological systems

Most telecaregiving interventions discussed in the literature seek to leverage technology to enable the remote delivery of care. Technological interventions fall into seven categories: (1) remote activity, lifestyle, and health monitoring; (2) reminder devices and memory aids; (3) emergency or assistance pendants and buttons; (4) video, messaging, and telephone communication; (5) localization and GPS tracking; (6) home security; and (7) telerobotics. Many ADRD telecaregiving interventions combine technologies from multiple categories, making them difficult to assign to a single category.

Table 1 defines these categories of technologies and subcategories thereof, citing mainly nonempirical literature on each in the ADRD caregiving domain. Table 2 summarizes the empirical studies on these technology interventions, including their study designs, study samples, and tested caregiver or care recipient outcomes.

**Table 1.** Technological Interventions for ADRD Telecaregiving

Intervention	Description
Remote activity, lifestyle, and health monitoring	Uses video or some other kind of sensor to monitor care recipients from afar (Behera et al., 2021; Bledsoe et al., 2010; Bowes et al., 2012; Davies et al., 2013; Faieta et al., 2021; Ganyo et al., 2011; Gillani & Arslan, 2021; Marling, 2007; Milligan et al., 2011; Pappadà et al., 2021; Stavropoulos et al., 2020; Wang et al., 2021; Wolff et al., 2021) •Most commonly discussed intervention in literature. •Some systems are “smart”: able to detect or predict health-related problems (Bowes et al., 2012)
Sensor alert systems	Remote monitoring using sensors (e.g., pressure, motion, environmental) (Behera et al., 2021; Davies et al., 2013; Gillani & Arslan, 2021; Marling, 2007; Milligan et al., 2011; Pappadà et al., 2021; Stavropoulos et al., 2020) •Used in variety of ways (e.g., appliance left on, where care recipient is in home, care recipient activities) •Preferred to video monitoring as it offers more privacy •Generally positive reactions from caregivers (Behera et al., 2021)
Fall detectors	Sensors specifically to detect if a care recipient has fallen (Behera et al., 2021; Ganyo et al., 2011; Stavropoulos et al., 2020; Wang et al., 2021) •Can be ambient or wearable sensors •Can offer caregivers and care recipients sense of security (Ganyo et al., 2011; Wang et al., 2021)
Video monitoring	Using video cameras to remotely monitor care recipients (Behera et al., 2021; Bledsoe et al., 2010; Davies et al., 2013; Marling, 2007; Pappadà et al., 2021; Wolff et al., 2021) •Increased intrusion into care recipient privacy •No empirical studies found
Physiological monitoring	Using health or physiological data (e.g., blood pressure, heart rate, etc.) to monitor a care recipient’s health and well-being (Behera et al., 2021; Faieta et al., 2021; Stavropoulos et al., 2020) •Increasingly popular due to increased availability of wearable sensors (e.g., FitBit; Faieta et al., 2021)
Reminder devices and memory aids	Interventions that act as cognitive prosthetics to support care recipients with memory impairments (Behera et al., 2021; Davies et al., 2013; Faieta et al., 2021; Gillani & Arslan, 2021; Marling, 2007; Milligan et al., 2011; Pappadà et al., 2021; Wolff et al., 2021) •Second most commonly discussed intervention in literature •Especially relevant to dementia where memory problems are a significant concern
Calendars and scheduling	Using a (usually digital) calendar to assist care recipients with scheduling (Davies et al., 2013; Marling, 2007; Pappadà et al., 2021) •Many pre-existing interventions that can be adapted to telecaregiving; convenient •Easily combined with other types of interventions •Some rely on strict schedules (may be limiting)
Automated pill boxes and medication reminders	Devices reminding care recipients to take medications at the appropriate time (some also record whether the medication was taken or not; Behera et al., 2021; Davies et al., 2013; Milligan et al., 2011; Pappadà et al., 2021) •Could lower caregiver stress (Davies et al., 2013); shown to improve medication adherence and improve hospitalization rates (Behera et al., 2021)
Task prompters	Intervention that guides participants step-by-step through the completion of a specific task (Pappadà et al., 2021)
Alarms	Often combined with other intervention types to alert care recipients to important information, tasks to be completed, events, etc. (Behera et al., 2021; Faieta et al., 2021)
Item locators	Devices attached to easily lost items (e.g., keys) to help users locate them (Behera et al., 2021; Davies et al., 2013)
Reminder apps	Smartphone applications available to assist with memory impairments (Faieta et al., 2021; Gillani & Arslan, 2021)
Facial recognition reminders	Cognitive prosthetics that use facial recognition to assist care recipients in recognizing friends, family, or other acquaintances (Pappadà et al., 2021) •Limited evidence to support effectiveness or endorse this intervention
Localization and GPS tracking	Technologies (largely using GPS) that assist caregivers in localizing their care recipients (Behera et al., 2021; Gillani & Arslan, 2021; Pappadà et al., 2021; Riehle et al., 2011; Stavropoulos et al., 2020; Wolff et al., 2021) •Generally, caregivers find systems useful, but contradictory studies find decreases in user confidence with daily use (Behera et al., 2021) •Valued by care recipients as it provides more freedom (Behera et al., 2021) •GPS on smartphones is pervasive •May create a false sense of security (does not eliminate all outdoor threats, e.g., traffic)
Geofencing	Combined with GPS/localization technology to alert when care recipient leaves a pre-defined “safe zone” (Behera et al., 2021) •Can mitigate wandering
Transportation assistance	Use of GPS technology to create a cognitive prosthetic to help care recipients travel from location to location (Riehle et al., 2011)
Video, messaging, telephone communication	Increased opportunities for caregivers to communicate with their care recipients using various technologies (telephone, text messaging, social media, videoconferencing, etc.; Behera et al., 2021; Davies et al., 2013; Faieta et al., 2021; Pappadà et al., 2021; Wolff et al., 2021; Zagaria, 2012) •Helps keep care recipient connected to friends and family •Allows caregiver to “check in” on care recipient
Telepresence robots	Special kind of video communication allowing caregivers to be “present” through the use of a mobile robot with a camera and video display. (Behera et al., 2021; Pappadà et al., 2021)
Personal message cards	Similar to greeting cards where caregivers can record personalized messages to their care recipients. (Evans et al., 2016)
Home security	Technologies designed to keep care recipients safe in their homes (Behera et al., 2021; Davies et al., 2013; Faieta et al., 2021; Marling, 2007; Milligan et al., 2011)
Controllers and switches	When combined with a sensor, could detect a problem (e.g., gas leak) and allow a caregiver to act remotely to remedy the situation (e.g., activate the gas shutoff'; Behera et al., 2021; Davies et al., 2013; Faieta et al., 2021; Marling, 2007; Milligan et al., 2011)
Smart locks	Allows for remote verification that a door is locked, remote lock/unlock of a door, and ensures that care recipients do not forget to lock doors (Davies et al., 2013; Faieta et al., 2021)
Lights and smart plugs	Ensures proper illumination to ensure care recipient safety (e.g., outdoor security lights, night lights to prevent falls; Davies et al., 2013; Faieta et al., 2021) •“Smart” technology can activate lights/ electronics when required
Cameras and video doorbells	Allows a caregiver or care recipient to remotely view parts of a home/property (Davies et al., 2013; Faieta et al., 2021)
Outdoor intercoms	Allows a caregiver or care recipient to remotely communicate with a person outside the home (e.g., asking who is at the door) (Vincent et al., 2002)
Telephone blockers	Blocks calls from numbers that aren’t on a recognized “safe list” (Behera et al., 2021) •Protection from scams
Emergency or assistance pendants and buttons	Wearable pendant/button to allow care recipients to call for assistance; may direct call to a call center, a caregiver or, in some cases, emergency services (Davies et al., 2013; Milligan et al., 2011; Stavropoulos et al., 2020) •Relatively “unintelligent” intervention •May be limited only to early stages of dementia as care recipients need to remember to wear/charge the device and press the button when needed (Milligan et al., 2011)
Telerobotics	Robotic systems that allow for caregivers to remotely assist their care recipients (e.g., robotic arms; Lv et al., 2020) •One paper found presenting the technology, but no empirical studies assessing outcomes

*Note*: ADRD = Alzheimer’s disease or other related dementias.

**Table 2.** Empirical Studies Using Technological Interventions for ADRD Telecaregiving (*n* = 27)

Citation	Intervention	Study	Sample	Period	Outcome
Arntzen et al. (2016)	Various assistive technologies for younger people with dementia (localization, reminder devices, alarms, calendars, med reminders, item locators, home security, smart locks)	Longitudinal	*N* = 12 younger people with dementia and their caregivers	Up to 19 months	Caregiver and care recipient experience
Holthe et al. (2018)		Interview	*N* = 12 younger people with dementia and their caregivers	3 weeks to 18 months	Caregiver experiences with use of technologies, usefulness of interventions
Ault et al. (2020)	Smart home system for nighttime wandering (RAM, SAS, home security, lights and smart plugs, reminder devices, alarms)	Pilot	*N* = 5 older adults with dementia who have episodes of wandering and their caregivers	12 weeks	Incidences of wandering/exits from the home, caregiver depression/anxiety, caregiver sleep quality
Balasubramanian et al. (2021)	Alexa Echo Show 8 (reminder devices, calendars, video communication)	Survey (pilot)	Survey responses: *n* = 44 patients with various conditions (including dementia) and *n* = 7 caregivers	2+ months	Device use and usefulness, care recipient independence, care recipient and caregiver stress, lifestyle habits, care recipient mental and social well-being (self-reported impacts—no validated instruments; report positive effects)
Bantry White et al. (2010)	Commercial tracker (localization, geofencing)	Interview	*N* = 10 caregivers of people with dementia using a commercial tracker	N/A	Caregiver use of system, system acceptability, perceived system impact
Boman et al. (2014)	Videophone (video communication)	Focus group	FG #1: *n* = 8 occupational therapists FG #2: *n* = 5 caregivers of people with ADRD FG #3: *n* = 2 people with ADRD FG #4: *n* = 5 caregivers of people with ADRD FG #5: *n* = 4 people with ADRD	N/A	Intervention design requirements
Boman et al. (2016)	All-in-one ICT device (RAM, SAS, FD, reminder devices, med reminders, alarms, calendars, emergency button, home security, controllers/ switches)	Focus group and mock-up evaluation	FG #1: *n* = 6 occupational therapists FG #2: *n* = 4 people with CI FG #3: *n* = 4 caregivers of people with CI Mock-up evaluation: *N* = 12 (*n* = 9 health care professionals, 2 people with CI, 1 caregiver)	N/A	Device functionality satisfaction (overall satisfaction with device, appreciation of one device with many functionalities)
Boyd, Evans, Cheston, et al. (2017)	Personalized digital prompter (reminder devices, prompter)	Technology evaluation	*N* = 12 participants with mild/moderate dementia and their caregivers	4 weeks	Care recipient/caregiver goal achievement, user experience
Boyd, Evans, Orpwood et al. (2017)		Exploratory study	*N* = 9 dyads of people with ADRD and their caregivers	N/A	Prompt format effectiveness (successful completion of tasks)
Evans et al. (2021)		Interview	*N* = 26 dyads of people with ADRD and their caregivers	4 weeks	User experiences, technology usefulness, ease of use
Harris et al. (2021)		Feasibility trial	*N* = 11 dyads of people with ADRD (not in residential care) and their caregivers	4 weeks	Care recipient/caregiver goal achievement, self-reported success in using the device
Cahill et al. (2007)	Various assistive technologies (reminder devices, calendars, item locators, home security, lights and smart plugs, controllers/switches, telephone communication)	Questionnaire	*N* = 20 people with ADRD and their caregivers/families	3 months	Intervention(s) usefulness, use of intervention(s), willingness to pay for products, desired design changes
Della Mea et al. (2020)	Low power communication infrastructure (Localization, geofence, RAM, FD)	Proof-of-concept	Prototype test by *N*1 = 2 authors “Small number of patients” carried wearable inside retirement home *N*3 = 7 social workers	N/A	Range of device/system, reliability of fall detection Fall detection based on free fall library does not adequately capture types of falls from older adults
Evan et al. (2016)	Personal message cards	Technology evaluation	*N* = 10 reviews (out of 24 cards issued)	N/A	Use of intervention, goal attainment
Fowler-Davis et al. (2020)	Digital plug (records when an event—e.g., appliance use—takes place; RAM, SAS)	Survey (pilot)	*N* = 30 people with ADRD and their caregivers	4 months	Care recipient frailty and well-being; caregiver well-being and burden; user experience (~half of care recipient and caregiver well-being declined, 17 care recipients improved or constant frailty, 18/30 caregivers decrease in burden)
Gaugler, McCarron, et al. (2019)	RAM	Focus group	*N* = 54 diverse caregivers and HCPs to people with ADRD (50% professional, 81.3% informal)	N/A	Overall perceptions of precision medicine in ADRD context (including RAM)
Gaugler, Zmora, et al. (2019)	Motion sensor RAM system (RAM, SAS, emergency button)	Survey (within RCT)	*N* = 132 people with ADRD and their caregivers (64 assigned to intervention)	6 months	Caregiver self-efficacy, sense of competence, distress/burden (no significant effect on caregiver outcomes); System acceptability, feasibility, perceived utility
Mitchell et al. (2020)		Survey/ interview (within RCT)	*N*1 = 30 people with ADRD and their caregivers in intervention condition (survey); *N*2 = 7 semi-structured interviews of caregivers	6 and 18 months	System utility and acceptability
Zmora et al. (2021)		Survey (within RCT)	*N* = 36 people with ADRD and their caregivers	6 months	Caregiver self-efficacy, sense of competence, burden, role captivity, role overload (no significant effect on caregiver outcomes); Perceived usefulness and ease of use of system
Lee & Chuang (2021)	Older adult and ADRD care service platform (RAM, SAS, FD, localization)	Proof-of-concept	*N* = 25 professional caregivers and *N* = 15 family caregivers	N/A	Caregiver satisfaction with use of the system (93% for family caregivers), assessment of usefulness of device, fall detection accuracy (94%).
Liu et al. (2017)	GPS system for people with ADRD (localization, geofencing)	Questionnaire and focus group	*N* = 45 dyads of people with ADRD and their caregivers (for questionnaire); FG: *N* = 15 caregivers and 9 other stakeholders (police, care management, social workers, etc.)	6–9 months	Device use behavior, technology acceptance, experience using intervention, device usefulness
Mahoney (2011)	WIN project (allowed caregivers to monitor care recipients while at work; RAM, SAS)	Meta-synthesis (one relevant study)	*N* = 27 dyads of caregivers and care recipients	N/A	Factors influencing the adoption of technology for remote monitoring of older adults’ daily activities.
Megges et al. (2017)	Localization system (localization, geofencing)	Prototype pilot	*N* = 17 dyads of people with ADRD and their caregivers	4 weeks	Device usability; ratings of device functions/features; caregiver burden (no change), perceived self-efficacy (no change); frequency of device use (infrequent), willingness to purchase prototype
Milne et al. (2014)	Localization system (localization, geofencing)	Feasibility study	*N* = 8 caregivers and 4 people with ADRD completed the study *N* = 15 professional stakeholders	6 weeks–6 months	Perceived utility; caregiver QoL, caregiver stress (only 5 completed at follow-up, no real changes); time spent searching for care recipients (estimated shorter but not verified); number of wandering episodes; admission to/days in professional care; hospital/doctor visits (feasible to measure, no control to compare); cost effectiveness (infeasible to measure)
Nishiura et al. (2021)	Electric calendar (reminder devices, calendars)	Cross-over RCT	*N* = 23 older adults (27 initially recruited with 20 of those having ADRD—unclear how many people with ADRD included in analysis)	12 weeks	Daily activities related to health care (improved after intervention period), cognitive function (improvement in cognitive function after intervention)
Øderud et al. (2015)	GPS intervention (localization)	Technology evaluation	*N* = 208 people with ADRD (50% with informal caregivers using GPS to locate, other 50% just HCPs)	1 week–2+ years	Experiences of caregivers and care recipients using intervention (overall positive)
Olsson et al. (2013)	Passive positioning alarm (localization, geofencing)	Qualitative pilot	*N* = 5 dyads of people with ADRD and their spouses	N/A	Experiences using intervention
Olsson et al. (2016)		Exploratory pilot	*N* = 11 people with mild dementia	N/A	Perceptions of people with ADRD on intervention when demonstrated to them
Pot et al. (2012)	GPS intervention (localization, telephone communication)	Survey (pilot)	*N* = 28 dyads of people with ADRD and their caregivers	3 months	Caregiver impression of device; device acceptability and usability (caregiver and care recipient); caregiver role overload (no change); care recipient worry (decreased worry); caregiver worry (decreased worry)
Rajasekaran et al. (2011)	Multisensory intervention device for anxiety and agitation in patients with CI (RAM, physio-monitoring)	Reliability test	*N* = 6 healthy volunteers	N/A	System reliability
Rowe et al. (2009)	Nighttime monitoring system (RAM, SAS)	Questionnaire and interview	*N* = 33 (*n* = 16 in experimental condition)	12 months	System reliability, caregiver satisfaction with system; care recipient nighttime injuries, exits from home
Vincent et al. (2002)	Various environmental control systems (reminder devices, emergency button, RAM, home security, smart locks, lights and smart plugs, intercoms)	Technology trial	*N* = 5 users with moderate CI or functional limitations and their caregivers	15 months	Caregiver burden (psychological but not physical burden eased), participant satisfaction, intervention effectiveness (improvement in care recipient ability to perform daily activities)
Zwierenberg et al. (2018)	Lifestyle monitoring system (RAM, SAS)	Interview and technology field study	*N* = 41 caregivers and *N* = 7 case managers (completed both interviews)	300 days	User experiences
Protocol paper					
Goodman-Casanova et al. (2019)	TV-based assistive service for people with CI (reminder devices, calendars, video communication, RAM, physio-monitoring)	RCT (protocol)	*N* = 240 dyads of people with CI and their caregivers (anticipated)	12 months	Caregiver/care recipient QoL, medication compliance and adherence, care recipient functional status, device utilization, caregiver burden; Usability of intervention.

*Notes*: ADRD = Alzheimer’s disease or other related dementias; CI = cognitive impairment; FD = fall detector; FG = focus group; GPS = global positioning system; HCP = healthcare professional; ICT = Information and Communications Technology; QoL = quality of life; RAM = remote activity monitoring; RCT = randomized controlled trial; SAS = sensor alert system; Study Period N/A = no longitudinal component to the study; WIN = worker interactive networking.

#### Remote activity, lifestyle, and health monitoring

Remote activity, lifestyle, and health monitoring technology interventions have been evaluated for system performance (accuracy, reliability, etc.), caregiver and care recipient experiences using the device (usability, utility, etc.), caregiver outcomes (burden, well-being, etc.), and care recipient outcomes (frailty, well-being, etc.) in 13 empirical studies. Most of these studies included at least one measure of user experience.

Most empirical studies reported that participants found the interventions worthwhile and useful. For example, Zwierenberg et al. (2018, pp. 200–201) reported that a remote activity monitoring system gave participants “a measure of peace” and caregivers who responded positively to using a remote activity monitoring system in a clinical trial described in Mitchell et al. (2020) reported that the system “provided useful information, promoted peace of mind, was easy to use, prevented health crises for the person with ADRD, and promoted the person with ADRD’s independent living” (p. 94). However, that same clinical trial reported no significant improvement in caregiver outcomes of self-efficacy, sense of competence, or distress after 6 months of the intervention (Gaugler, Zmora, et al., 2019; Zmora et al., 2021).

Fowler-Davis et al. (2020) implemented a sensor alert system (a digital plug to detect timed events like turning on an appliance or light) with 30 people living with dementia and their caregivers and found approximately half the participants (both caregivers and care recipients) reported declines in their quality of life, in 77% of care recipients, frailty did not change or worsened, and 60% of caregivers reported improved caregiver burden compared to 33% who reported worse caregiver burden. Although Fowler-Davis et al. (2020) commented on the potential of the digital plug to lower caregiver burden (perhaps by offering peace of mind), there was no explanation provided as to the decline in quality-of-life measures. None of the reviewed studies demonstrated statistically significant effects.

In some studies, fall detection systems were shown to give caregivers and care recipients a sense of security (Ganyo et al., 2011), but there is conflicting empirical evidence regarding the accuracy and reliability of these systems for identifying the kinds of falls older adults experience (Della Mea et al., 2020; Lee & Chuang, 2021).

### No Empirical Papers Reported Studies of Video Monitoring Systems

#### Reminder devices and memory aids

Most of the nine empirical studies of reminder devices and memory aids examined user experience or intervention performance outcomes and results tended to be favorable. Among studies measuring caregiver and care recipient outcomes, Vincent et al. (2002) demonstrated a positive impact on psychological caregiver burden, but no physical burden, and Balasubramanian et al. (2021) reported using a smart speaker intervention positively affected care recipient health and well-being. Ault et al. (2020) implemented caregiver recording alarms to guide participants back to bed if they were wandering at night as part of a nighttime wandering monitoring system. The system demonstrated decreases in caregiver anxiety and depression among all five study participants and increases in sleep quality for three participants (the other two participants attributed their constant or decreased sleep quality to factors outside of the system). No study demonstrated statistically significant effects for caregivers or care recipients.

#### Localization and GPS tracking

There are 10 empirical studies investigating localization and GPS tracking technologies for people with ADRD and many reported overall positive perceptions of tracking interventions from both caregivers and care recipients (Lee & Chuang, 2021; Øderud et al., 2015; Olsson et al., 2013, 2016). Liu et al. (2017) reported high acceptance of their localization system and an overall impression that dyads were likely to continue to use the device. In Bantry White et al. (2010), most caregivers preferred to use a GPS tracking system as a backup to other strategies of care (e.g., locked doors or caregiver supervision). In cases where caregivers perceived the risk of their care recipient getting lost to be low, the GPS system preserved care recipient independence (Bantry White et al., 2010).

Studies found that tracking technologies enhanced perceptions of independence for both caregivers and care recipients and gave both parties a sense of reassurance (Bantry White et al., 2010; Liu et al., 2017; Pot et al., 2012). In Milne et al. (2014), caregivers estimated that they spent less time searching for care recipients, though this was not verified by observation.

Three studies assessed caregiver outcomes including burden or stress, role overload, quality of life, and perceived self-efficacy using validated measures (Megges et al., 2017; Milne et al., 2014; Pot et al., 2012). None of these demonstrated statistically significant effects of the intervention on any caregiver outcome.

#### Video, messaging, and telephone communication

Six empirical studies investigated the use different forms of communication between caregivers and care recipients. There is evidence that communication between care recipients and caregivers can improve their social connectedness with one another (Balasubramanian et al., 2021; Evans et al., 2016). Balasubramanian et al. (2021) investigated the use of a smart speaker with a screen and videoconferencing functionality and concluded that “users felt that the technology brought the family members closer and even facilitated bonding across generations” (p. 6). Similarly, Evans et al. (2016) demonstrated personal message cards were viewed positively by family members as a way to connect to loved ones with dementia and could have benefits even for patients with more severe dementia.

Boman et al. (2014) outlined the design requirements for a videophone for people with ADRD to enable them to more easily connect to others, but did not test its effects. Cahill et al. (2007) provided a picture button telephone as part of an intervention suite that allowed both caregivers and care recipients to push buttons with the picture of the person they wished to call instead of having to remember phone numbers. This was viewed positively by caregivers for both care recipients and themselves.

Pot et al. (2012) added a calling ability to their GPS tracking intervention to enable caregivers and care recipients to call each other. Those caregivers that used this feature and were able to reach care recipients over the phone reported increased improvements in their feelings of worry, but no statistically significant change in role overload. However, a subset of caregivers expressed concerns that care recipients would not know the origin of voice calls made through the GPS device and this disembodied voice would cause distress.

#### Home security

Home security interventions were investigated in five empirical studies. Arntzen et al. (2016) and Holthe et al. (2018) offered a smart door lock to a single participant but did not report its effects on any outcomes. An automatic lamp in Cahill et al. (2007) was considered somewhat useful by participants but was not used often; the gas cooker monitor in the same study had major technical difficulties and changed the appearance of the stove, leading participants with ADRD to abandon its use. Vincent et al. (2002) tested multiple home security interventions with five participants and their caregivers and found that participants did not consider smart locks, intercoms, and stove shut-off interventions to be efficient, whereas automatic lights or nightlights functioned as intended, despite being susceptible to accidental activation by pets. Ault et al. (2020) had lights turn on as part of their nighttime wandering monitoring system to guide participants with dementia to the bathroom when they got up in the night. These lights functioned as intended and the system as a whole had positive impacts on caregiver anxiety and depression.

Focus group participants in Boman et al. (2016) listed automatic shut offs for home equipment as a design requirement for an all-in-one device but such a feature was not developed and tested with participants.

#### Emergency or assistance pendants and buttons

No empirical study had a sole or major focus on emergency/assistance pendants or buttons but they were evaluated as part of other interventions in two different empirical studies. Vincent et al. (2002) demonstrated an improvement in the psychological aspects of caregiver burden in a study of multiple technologies including an emergency pendant.

When considering caregiver experiences and perceptions of emergency or assistance pendants and buttons, the ability to have a care recipient call for help was seen as very important by many (Boman et al., 2016) and it was shown in Vincent et al. (2002) that emergency/assistance pendants or buttons can increase feelings of safety among caregivers and care recipients, even if the device never has to be used.

#### Telerobotics

Currently, there is no empirical evidence supporting telerobotic use for ADRD telecaregiving. Lv et al. (2020) described and demonstrated the lab-based performance (e.g., motion accuracy) of a dual-arm telerobotic system that would enable caregivers to remotely assist people living with ADRD in the performance of household tasks and enhance care recipient mobility. The robotic system used motion capture technology to have the robot mimic the movements of the operator.

#### Ongoing technological intervention trials

Two studies identified in this review were ongoing randomized controlled trials (RCTs).

Goodman-Casanova et al. (2019) reported their RCT protocol for testing a television-based intervention with videoconferencing, reminder, calendar, and physiological monitoring capabilities to support patients with mild cognitive impairment or mild dementia. The goal of this ongoing RCT of 240 caregiver–care recipient dyads is to assess the effect of the intervention on caregiver and care recipient quality of life, care recipient medication compliance and adherence, care recipient functional status, and caregiver burden.

Gaugler, Zmora, et al. (2019), Mitchell et al. (2020), and Zmora et al. (2021) presented mid-study results from an ongoing RCT to test a remote activity monitoring system combined with an emergency pendant. To date, no statistically significant results have been reported on the effect of the intervention on study-assessed caregiver outcomes such as burden, role captivity, role overload, and sense of competence.

### Nontechnological Interventions

Although most interventions in the literature relied on technology to bridge the gap between physically distanced caregivers and care recipients with ADRD, several categories of nontechnological interventions exist (Table 3) and have been the subject of empirical studies (Table 4): (a) formal care services; (b) organizational long-distance caregiver programs; and (c) fall prevention checklists.

**Table 3.** Nontechnological Interventions for ADRD Telecaregiving

Intervention	Description
Formal care services and professionals	The hiring/use of professional caregivers (e.g., home aids, care homes, home nurses) to provide care to the care recipient (Koerin & Harrigan, 2003; Lewis, 2008; Zagaria, 2012) •Valuable resource for many caregivers of people living with dementia
Organized long-distance caregiver programs	Los Angeles Alzheimer’s Association long-distance caregiver program for caregivers outside the county caring for someone living within the county. It consisted of (1) consult with professional family consultant, (2) printed or web-based resource guides, (3) access to informational website, (4) telephone legal consult, (5) financial and care planning services, and (6) program to help find and return lost or wandering care recipients (Bledsoe et al., 2010; Watari et al., 2006)
Fall prevention checklists	List addressing changes that can be made in each room of a home to minimize the risk of falls (Zagaria, 2012)

*Note*: ADRD = Alzheimer’s disease or other related dementias.

**Table 4.** Empirical Studies of Nontechnological Interventions for ADRD Telecaregiving (*n* = 4)

Citation	Intervention	Study	Sample	Outcome
Falzarano et al. (2020)	Formal care services	Interview	*N* = 296 long-distance caregivers: *n* = 58 with care recipients with dementia in residential care, *n* = 49 care recipients with dementia in community	Long-distance caregiver experience (satisfaction and challenges) with formal care providers
Falzarano et al. (2022)		Interview	*N* = 166 (28.7% cared for someone with dementia) caregivers	Caregiver strain (home care services fully mediate relationship between care recipient functional status and caregiver strain; partially mediate relationship between care recipient cognitive status and caregiver strain)
Giebel et al. (2021)	Formal care services	Interview	*N* = 16 unpaid dementia caregivers	Caregiver experiences accessing care and services during COVID-19
Gunn et al. (2021)	Formal care services	Interview	*N* =13 adult long-distance caregivers for older adults	Factors influencing choice of formal care provider, experiences with formal care provider
Watari et al. (2006)	Organized long-distance caregiver programs	Survey	*N* = 90 long-distance caregivers and *n* = 187 local caregivers of people with ADRD	Services used, satisfaction with services (long-distance caregivers had higher satisfaction)

*Notes*: ADRD = Alzheimer’s disease or other related dementias; COVID-19 = coronavirus disease 2019.

#### Formal care services and professionals

For many caregivers who are temporarily or permanently distanced from a care recipient with ADRD, formal care services such as home aides, care homes, and hiring of professional caregivers may provide care support when needed. Formal care services were investigated in three empirical studies. Falzarano et al. (2022) investigated the effect of formal care services on long-distance caregiver strain. They reported that care recipient cognitive and functional status were significantly associated with increased interference with caregivers’ family responsibilities, but that caregivers experienced a less associated increase in family interference when they utilized home care services.

Studies of remote caregivers (both long-distance caregivers and proximal caregivers not living with care recipients) report cost and local availability of services as a significant barrier (Giebel et al., 2021; Gunn et al., 2021). There may also be limited availability of care services at the time when the caregiver needs them (Giebel et al., 2021; Gunn et al., 2021); this problem was exacerbated during the coronavirus disease 2019 pandemic when accessing care homes was more difficult or impossible and visitation for informal caregivers was severely limited or prohibited (Giebel et al., 2021).

#### Organized long-distance caregiver programs

One reviewed empirical study investigated the Los Angeles Alzheimer’s Association long-distance caregiver program (Watari et al., 2006). This study examined whether long-distance versus proximal caregivers used different services in the program and each group’s satisfaction with the services. There was no significant difference in the services used between groups but long-distance caregivers expressed higher satisfaction with those services (Watari et al., 2006).

#### Fall prevention checklists

Although Zagaria (2012) recommended that informal caregivers receive a Falls Prevention Home Safety Check List, this intervention had not been empirically studied as a system to support ADRD telecaregiving.

### RQ3: Outcomes of Systems to Support ADRD Telecaregivers

Table 5 presents the empirical outcomes evaluated in studies included in this review. Table 5 also includes outcomes that were not systematically measured but were mentioned as future measurements or were assessed in some other way (e.g., feasibility assessments). We say that an outcome was “systematically assessed” if it was measured using a replicable method and if that measurement could be compared pre- and postintervention. If an outcome is listed as systematically assessed in Table 5, that does not mean every study assessing that outcome did so systematically (e.g., some studies assessed user experience in a systematic way, whereas others did not), but that at least one study did.

**Table 5.** Studied Outcomes of ADRD Telecaregiving Interventions

Outcomes systematically assessed in studies	Outcomes mentioned but not systematically assessed
•User experience •Use of intervention •Intervention design requirements •Intervention performance •Caregiver outcomes ◦Burden/stress ◦Role overload/captivity ◦Well-being/quality of life ◦Self-efficacy/competence ◦Goal attainment ◦Time requirements ◦Sleep quality •Care recipient outcomes ◦Independence ◦Well-being/quality of life ◦Frailty ◦Health activity ◦Cognitive status ◦Functional status ◦Stress ◦Hospitalization/injuries ◦Wandering episodes ◦Goal attainment	•Intervention cost effectiveness •Care recipient falls

*Note*: ADRD = Alzheimer’s disease or other related dementias.

Most studies assessed user experience outcomes. When other caregiver or care recipient outcomes were evaluated, the most frequently used were those related to the psychosocial impact on the caregiver (e.g., burden, strain, overload) and care recipient independence (e.g., number of tasks they can complete unaided). Only one study reported a significant effect on caregiver or care recipient outcomes: Falzarano et al. (2022) found that the increase in caregiver stress associated with declines in care recipient functional and cognitive status was minimized when home care services were utilized. Although it is possible that the lack of statistically significant findings indicates that these interventions do not have effects on care recipient or caregiver outcomes, it is likely attributable to the small sample sizes (*N* = 5–36, see Table 2) in most of the empirical studies in this review (though a small number of studies with larger sample sizes may indicate the studied intervention has no effect on these outcomes, e.g., Gaugler, Zmora, et al., 2019).

## Discussion and Implications

In general, telecaregiving has not received much attention in the caregiving literature. Various types of caregivers can be telecaregivers at different points in time from long-distance caregivers living hours away to caregivers in the same household who want peace of mind through unobtrusive check-ins on care recipients. However, most of the literature describing the experiences of telecaregivers focuses only on long-distance caregivers, and data describing this study population are dated. Additionally, only one older case study (Collins et al., 2003) investigated how long-distance caregiving experiences may differ among different demographic groups. This highlights not only the need to update our understanding of the current nature of long-distance caregivers, but to also investigate the diversity of telecaregiver types and to compare telecaregiver needs between sociodemographic groups.

Even considering long-distance caregivers specifically, the literature explicitly identifies the lack of attention they have received: Much has been written about the negative impacts of caregiving without addressing distance specifically, and long-distance caregivers are virtually absent from intervention efforts to mitigate caregiver strain (Alzheimer’s Association, 2013; Bledsoe et al., 2010; Falzarano et al., 2020, 2022; Koerin & Harrigan, 2003). In ADRD telecaregiving specifically, this exclusion from the literature is even more apparent; many of the studies on telecaregiving that do exist are not exclusive to caregivers of people with ADRD, limiting our understanding of the specific needs of this population.

Without a clear and thorough understanding of telecaregivers’ needs, it is unlikely that interventions will be developed that adequately address those needs. We, therefore, propose that *future work must begin by more deeply understanding the current nature of telecaregiving* to enable the development of effective telecaregiving interventions. We recommend the use of human factors or user needs analyses such as contextual inquiry to create telecaregiver/care recipient personas and journey maps (Werner et al., 2022), decision-making models (Daley et al., 2018), workflows (Mickelson et al., 2016; Ozkaynak et al., 2018; Unertl et al., 2010), or task analyses (Holden et al., 2020).

There is a question as to whether the interventions currently developed to support telecaregivers adequately address general and ADRD-specific telecaregiving needs to be discussed in the literature. Additionally, sources in this mapping review discussed the lack of thorough user testing done by companies producing assistive technologies, and the lack of involvement of people with ADRD and ADRD caregivers early in the development stage (Behera et al., 2021; Zmora et al., 2021). In this review, many of the studies explored user experience of their interventions (often asking participants open-ended, retrospective questions or utilizing usability surveys such as the System Usability Scale) and some sought stakeholder involvement in the design process (e.g., Tiersen et al., 2021). However, there were no studies that employed more in-depth user-centered design methodologies such as participatory design (Reddy et al., 2019; Werner et al., 2022) specifically with telecaregivers, and few employed more comprehensive usability testing in the evaluation of their interventions. We, therefore, propose that researchers employ user-centered design and evaluation techniques to design and test interventions for their ability to meet telecaregiver users’ needs. User-centered design and user testing with diverse groups of users should be an integral part of the development of any telecaregiving technology, especially those marketed toward people living with ADRD and ADRD caregivers.

Work on telecaregiving interventions is rarely accompanied by empirical evidence to support their efficacy (Bledsoe et al., 2010). Of the empirical studies that have been performed, the sample sizes are generally small and may be underpowered to detect statistical or clinically meaningful effects. Small sample sizes also limit subgroup analyses, impeding the identification of groups that differentially benefit from telecaregiving interventions (Davies et al., 2013; Gaugler, Zmora, et al., 2019).

Several outcomes related to telecaregiving were investigated in the literature. Most studies included user experience outcomes and reported participants having favorable opinions of interventions. This is useful in terms of refining and improving a system and generally determining its acceptability to users but does not help determine an intervention’s effectiveness in addressing care recipient or caregiver outcomes such as stress, symptom burden, or quality of life.

Additionally, there are many outcomes that have not been considered in the literature, such as measures of the effects of interventions on behavioral and psychological symptoms of dementia (BPSD), the quality of care received by care recipients, accessibility or sustainability of interventions, the reach and diversity of communities served by interventions, the cost–benefit analysis of proposed interventions, and the effect of interventions on health equity. It is also clear that caregiver outcomes have been prioritized over care recipient outcomes. This is not unexpected as most of the studied interventions target the caregiver. We, therefore, recommend that *future studies of ADRD telecaregiving investigate intervention affects on both caregivers and care recipients*.

It is difficult to conclusively determine if the interventions that have been proposed and tested in this review have positive, negative, or no effect on clinical outcomes due to the limited nature of clinical outcome data in these studies. Therefore, we recommend that larger-scale studies should be performed on both new and existing interventions to systematically assess their effects on a variety of key caregiver and care recipient outcomes. Based on the favorable user experiences reported in many studies, we believe that there is merit in performing larger-scale studies on promising existing interventions to generate sufficient data to determine their impact on caregivers and care recipients.

### Methodological Considerations

This review study had some limitations. To more completely map the work done in the area, studies of any design from both peer- and nonpeer-reviewed sources were included, and the quality of the included studies was not assessed. Therefore, studies of varying quality were included and future studies should target not only gaps in the literature but areas needing greater research rigor. Additionally, the inclusion of a variety of sources and study types means that the information extracted from the included papers is heterogeneous, making synthesis and comparisons across studies difficult or impossible. The search keywords were also not tailored to each database.

There is a lack of existing, standardized vocabulary in this research area, raising the risk of relevant studies being excluded. Various strings of keywords were generated to attempt to find an ideal balance between the inclusion of many kinds of studies relevant to this review while minimizing irrelevant sources. For example, we considered creating a string of “technology” keywords, as part of the goal of this review was to assess the different types of interventions used to support ADRD telecaregiving, many of which are technology based. However, due to the large number of different types of technologies and the numerous keywords describing different types of technology, we were not confident we would compile a comprehensive list of all possible types of technological interventions and did not want to exclude a type of technology through omission. We, therefore, did not explicitly include “technology” in our keyword search, though researchers seeking papers in this area may benefit from the inclusion of some of these technology keywords relevant to their research goals.

The 19 papers found outside of our keyword searches imply that despite our very broad keywords, the literature in this area is not yet very conducive to systematic review. This exposes the need for more standardization in the keywords used to describe this work. In the way “telemedicine” was able to unify otherwise heterogeneous work done in the remote delivery of health care, we hope that the term “telecaregiving” as established in this review can act as a keyword to unify the large variety of work done in many different fields under the same umbrella. In fact, it is our view that the next decade may present opportunities that enable this term to be widely adopted in the research and health care community owing to political and economic incentives (e.g., infrastructure legislations that boost access to broadband internet) that create fertile ground for design and deployment of innovative telecaregiving interventions. As a result, it would be easier for researchers to remain informed of new telecaregiving developments and interventions.

The target of this review was on ADRD telecaregiving and literature on telecaregiving or remote caregiving in other areas (e.g., diabetes management) not reviewed here could provide additional insight relevant to ADRD telecaregiving.

## Conclusion

The literature conceptualizes modern telecaregiving as the delivery of care from shorter and longer distances with the support of innovative technological and nontechnological interventions whose primary users are unpaid, informal caregivers. These telecaregiving interventions are a new frontier of telehealth and telecare with a high potential to improve outcomes for caregivers, care recipients, and others. However, this review revealed three major knowledge gaps requiring further research:

1. We do not fully understand the needs and day-to-day activities of ADRD telecaregiving;
2. Interventions developed to support ADRD telecaregiving may not fully meet the needs of caregivers or care recipients; and
3. There is insufficient rigorous research establishing intervention effectiveness on key ADRD care recipient and ADRD caregiving outcomes (e.g., caregiver burden, BPSD, falls, hospitalizations, etc.).

Future work in telecaregiving should seek to address the three gaps identified. This includes more studies taking a user-centered approach to understand and design interventions for a larger variety of ADRD telecaregivers and their needs, followed by rigorous tests of these interventions.

## Supplementary Material

gnad026_suppl_Supplementary_Data

gnad026_suppl_Supplementary_Material

## Data Availability

Summaries of all included studies and the data extraction tool used in this review are available as supplementary materials online from The Gerontologist. This review was not pre-registered.
