# Supplementary material for: Telecaregiving for Dementia: A Mapping Review of Technological and Nontechnological Interventions
Source: Gerontologist. 2023 Mar 15;64(1):gnad026. doi: 10.1093/geront/gnad026 (PMC10733214; doi:10.1093/geront/gnad026)
Supplement: gnad026_suppl_Supplementary_Material [file gnad026_suppl_supplementary_material.docx]

**Online Supplementary Material**

## Summary of Included Sources

| **Study** | **Citation** | **Source Type** | **Study Design** | **Target Population** | **Intervention(s):** Discussed  **Tested** | **Sample Size** | **Tested Outcomes** | **Brief Description** |
| --- | --- | --- | --- | --- | --- | --- | --- | --- |
| **Studies** | | | | | | | | |
| 1 | Arntzen et al., 2016 | Journal Article | Qualitative Longitudinal Study | Younger people with dementia (<66) and their caregivers | **Localization & GPS tracking; reminder devices & memory aids; alarms; calendars/ scheduling; automated pill boxes/med reminders; item locators; home security; automatic/ remote locks** | N=12 younger people with dementia and their caregivers | Caregiver/care recipient experience implementing assistive technology (AT)  Identified factors important for the experience of usefulness and successful incorporation of AT | Follows 12 younger people with dementia and their caregivers as they implement 1+ AT into their lives |
|  | Holthe et al., 2018 | Journal Article | Qualitative Interview Study | Younger people with dementia (<66) and their caregivers | **Localization & GPS tracking; reminder devices & memory aids; alarms; calendars/ scheduling; automated pill boxes/med reminders; item locators; home security; automatic/ remote locks** | N=12 younger people with dementia and their caregivers | Caregiver experiences of the use of AT, potential benefits/burdens of AT, effects of intervention implementation, usefulness of interventions | Repeated semi-structured interviews with caregiverss of people living with dementia about their use of AT |
| 2 | Ault et al., 2020 | Conference Proceeding | Mixed-Methods Pilot Study | Older adults with dementia and their caregivers | **Remote activity, lifestyle and health monitoring; sensor alert systems; home security; lights and smart plugs; reminder devices & memory aids; alarms** | N=5 older adults with dementia who wander and their caregivers | Incidences of wandering/exits from the home, caregiver anxiety/depression (decreased), caregiver sleep quality (decreased). | Installed an off-the-shelf nighttime wandering monitoring system in 5 homes. Ongoing pilot. |
| 3 | Balasubramanian et al., 2021 | Journal Article | Qualitative Pilot Study | Patients with various conditions (including dementia) and their caregivers | **Reminder devices & memory aids; video communication; calendars/ scheduling** | n=44 patients  n=7 caregivers  Survey Responses  (unspecified # of people living with dementia)  Focus groups of n=23 and n=4 (diabetes patients only) | Self-reported outcomes. Device usefulness; care recipient independence, care recipient & caregiver stress, lifestyle habits (diet & exercise), care recipient mental and social well-being.  Overall reported positive caregiver and patient experience with device; majority said voice control made it easier to use; reported useful for organizing lives of patients, relieved stress for caregivers; reported positive impact on mental and social well-being | Describes user experience of Amazon Echo Show (smart speaker) to improve health and wellbeing. |
| 4 | Bantry White et al., 2010 | Journal Article | Qualitative Interview Study | People living with dementia and their CGs | **Localization & GPS tracking; geofencing** | N=10 caregivers of people living with dementia using a commercial tracker | Caregiver use of system, system acceptability, system impact.  Most thought tech was accessible, some thought device was too large. Thought device should look like a familiar object (e.g., watch). Most use system as backup to other interventions/supervision. 9/10 thought it gave peace of mind, some thought it enhanced care recipient independence and freedom. | Qualitative interview with caregivers of people living with dementia who use GPS tracking intervention |
| 5 | Behera et al., 2021 | Journal Article | Systematic Review | People living with dementia and their caregivers | Reminder devices & memory aids; item locators; automated pill boxes; alarms; video communication; telepresence robots; localization & GPS tracking; geofencing; remote activity, lifestyle, and health monitoring; sensor alert systems; fall detectors; video monitoring; physiological monitoring; home security; controllers & switches; telephone blockers | N/A | N/A | Describes assistive technologies for dementia |
| 6 | Bledsoe et al., 2010 | Journal Article | Evaluative Review | Long-distance caregivers of elderly relatives living outside institutions | Remote activity, lifestyle, and health monitoring; video monitoring; organization developed or led long-distance caregiver programs | N/A | N/A | Reviews empirical research to identify gaps in knowledge regarding long-distance caregivers |
| 7 | Boman et al., 2014 | Journal Article | Qualitative Focus Group Study | People living with dementia | **Video communication** | FG #1: n=8 occupational therapists  FG #2: n=5 caregivers to people with dementia  FG #3: n=2 people with dementia  FG#4: n=5 caregivers to people with dementia  FG#5: n=4 people with dementia | Intervention design requirements | Develops a design concept for an easy-to-use videophone for people with dementia |
| 8 | Boman et al., 2016 | Journal Article | Scoping Review/  Focus Group Study/ Mock-Up Evaluation (Qualitative) | Individuals with cognitive impairments and their caregivers and healthcare professionals | **Reminder devices & memory aids; automated pill boxes; alarms; calendars/ scheduling; emergency/assistance pendants/buttons; remote activity, lifestyle, and health monitoring; sensor alert systems; fall detectors; home security; controllers & switches** | FG #1: n= 6 occupational therapists  FG #2: n=4 persons with cognitive impairment  FG #3: n=4 Caregivers of persons with cognitive impairment  Mock-up evaluation (N=12): n=9 healthcare professionals, n=2 persons with cognitive impairment, n=1 caregiver | Self-reported outcomes.  Overall satisfaction with functionalities of mock-up; calendar most important functionality but interface was too complex for persons with cognitive impairment; some design improvement and additional feature suggestions provided; participants liked the monitoring aspects; participants liked that it was an all-in-one device | Identifies target population’s needs and requirements of an all-in-one ICT-based device; evaluation of first device mock-up. |
| 9 | Bowes et al., 2012 | Journal Article | Review/ Discussion | Ageing researchers, older adults and their caregivers | Remote activity, lifestyle, and health monitoring | N/A | N/A | Identifies and discussed ethical issues surrounding third-generation telecare systems. |
| 10 | Boyd, Evans, Cheston, et al., 2017 | Conference Proceeding | Mixed-Methods Technology Evaluation | People living with dementia and their caregivers | **Reminder devices & memory aids; task prompters** | N=12 participants with mild/moderate dementia and their caregivers | Self-reported outcomes. Semi-structured interviews. Goal achievement (if dyads met the goals they set at the beginning of the project), user experience  All people living with dementia able to use prompter, many thought it was useful. 10/24 care recipient goals achieved, 6/24 partially achieved. 8/24 not achieved. 3 caregiver goals achieved, 2 partially, 7 not achieved. | Presents participant views on a personalized digital prompter. |
|  | Boyd, Evans, Orpwood, et al., 2017 | Journal Article | Quantitative Exploratory Study | People living with dementia and their caregivers | **Reminder devices & memory aids; task prompters** | N=9 dyads of people living with dementia and caregivers | Prompt format effectiveness (determined by successful completion of tasks) | Paper explores different task prompt designs/formats to determine the most efficacious method |
|  | Evans et al., 2021 | Journal Article | Qualitative Interview Study | People living with dementia and their caregivers | **Reminder devices & memory aids; task prompters** | N=26 dyads of people living with dementia and their caregivers | Experiences of people living with dementia and caregivers using the intervention, technology usefulness, technology ease of use | Interview of people living with dementia and their caregivers after using a digital prompter for 4 weeks |
|  | Harris et al., 2021 | Journal Article | Quantitative Feasibility Trial | People living with dementia and their caregivers | **Reminder devices & memory aids; task prompters** | N=11 (originally 14 but 3 withdrew) dyads of people living with dementia not in residential care and their caregiver | Goal achievement (if dyads met the goals they set at the beginning of the project), success in using the device.  14/22 dyad goals achieved, 1 partially, 7 not met. | Feasibility trial of a personalized digital prompter. |
| 11 | Cahill et al., 2007 | Journal Article | Mixed-Methods Questionnaire Study | People living with dementia and their caregivers | **Reminder devices & memory aids; calendars/ Scheduling; item locators; home security; lights & smart plugs; controllers & switches; telephone communication** | N=20 people living with dementia and their caregivers/families | Usefulness of interventions, use of interventions; willingness to pay for products; desired design changes | Papers explores use and usefulness of different AT (provided by the study) for people living with dementia |
| 12 | Collins et al., 2003 | Journal Article | Case Study | African American family involved in long-distance caregiving of a person with Alzheimer’s disease. | N/A | N/A | N/A | Presents a case study to discuss challenges of long-distance caregiving and present strategies to lessen caregiver stress. |
| 13 | Davies et al., 2013 | Journal Article | Systematic Review | People with social care needs and their caregivers | Reminder devices & memory aids, item locators; automated pill boxes; calendars/scheduling; emergency/assistance pendants/buttons; video, messaging, telephone communication; remote activity, lifestyle, and health monitoring; sensor alert systems; video monitoring; home security; cameras & video doorbells; automatic/remote door locks; controllers & switches; lights & smart plugs | 3 (out of 7) studies relevant to dementia.  N1=25  N2=16  N3=126 | caregiver stress (N1 & N3), descriptive carer outcomes (N2, free time, relationship with care recipient, etc.)  N1 & N3 showed statistically significant reduction in caregiver stress. N2 showed improvements in freeing up caregiver time, affected relationships with care recipient s, made life easier, negative effects | Evaluates quantitative evidence base for the effect of telecare intervention on outcomes for caregivers |
| 14 | Della Mea et al., 2020 | Journal Article | Mixed-Methods Proof-of-concept | People living with dementia and their caregivers | **Localization & GPS tracking; geofencing; remote activity, lifestyle, and health monitoring; fall detectors** | Prototype test performed by two authors (N1=2)  “Small number of patients” (N2=??) carried wearable inside retirement home and nurses access the web platform  N3=7 social workers carried around wearable to map gateway coverage | Range of device/system.  Max distance from gateways 30.5km (N1); fall recognition not reliable, GPS not able to provide position when device far from windows (N2); node communication radius 12km (N3) | Describes infrastructure designed to support IoT system deployment to support independence of people living with dementia; presents the results of 3 experiments. |
| 15 | Evans et al., 2016 | Journal Article | Qualitative Technology Evaluation | People living with dementia and their caregivers | **Personal message cards** | N=10 reviews of cards | Use of intervention, goal attainment | Tech resource for people living with dementia and caregivers issued personal message cards for use and analyzed reviews of the technology |
| 16 | Falzarano et al., 2020 | Journal Article | Mixed-Method Interview Study | Long-distance caregivers of functionally impaired older adults | **Hiring Formal Care Services/Homes/ Professionals** | N=296 long-distance caregivers: n=58 care recipient s in residential care with dementia, n=65 care recipient s in residential care with no dementia, n=49 care recipient with dementia in the community, n=144 no dementia in the community | LDC experience (satisfaction and challenges) with formal care providers. Challenges related to formal care significantly greater and satisfaction significantly lower among long-distance caregivers of care recipient s in residential care, irrespective of dementia status when compared to long-distance caregivers of care recipient s in the community. | Documents long-distance caregiver experiences with formal care and measures their satisfaction |
|  | Falzarano et al., 2022 | Journal Article | Mixed-Method Interview Study | Long-distance caregivers of community-dwelling older adults | **Hiring Formal Care Services/Homes/ Professionals** | N=166 (28.7% cared for someone with dementia) | Caregiver strain (home care fully mediates association between functional impairment and caregiver strain and partially mediates effects of cognitive impairment on caregiver strain) | Investigates whether association between primary caregiving stressors and secondary stressors in long-distance caregivers is mediated by the use of home care services. |
| 17 | Fowler-Davis et al., 2020 | Journal Article | Mixed-Methods Pilot Study | People living with dementia and their caregivers | **Remote activity, lifestyle, and health monitoring; sensor alert systems** | N=30 people living with dementia and their caregivers | Care recipient frailty and wellbeing; caregiver wellbeing & burden  Care recipient s reported decline in wellbeing and increased frailty. Caregivers reported decline in wellbeing but some reported a reduction in burden (n=18) | Pilot study implemented a digital plug into homes of people living with dementia to help caregivers track repeated activities (e.g., everytime kettle turns on) |
| 18 | Ganyo et al., 2011 | Journal Article | Review/ Discussion | Older adults & those susceptible to falls | Remote activity, lifestyle, and health monitoring; fall detectors | N/A | N/A | Outlines currently available fall detectors and discusses ethical issues associated with them |
| 19 | Gaugler, McCarron, et al., 2019 | Journal Article | Qualitative Focus Groups Study | Diverse caregivers and healthcare professionals to people living with dementia | **Remote activity, lifestyle, and health monitoring** | N=54 participants, 50% care for person with memory loss in professional capacity, 81.3% informal caregivers to person with memory loss | Participants’ experiences related to ADRD care, perceived challenges, gaps in care, overall perceptions of precision medicine in ADRD context. | Investigates how diverse family caregivers and healthcare professionals view precision medicine interventions for dementia care. |
| 20 | Gaugler, Zmora, et al., 2019 | Journal Article | Mixed-methods Survey Study (within RCT) | Persons with ADRD and their caregivers | **Remote activity, lifestyle, and health monitoring; sensor alert systems; emergency/assistance pendants/buttons** | N=132 people living with dementia and their caregivers (n=64 randomly assigned to RAM intervention) | Care recipient dependence on assistance with ADLs and IADLs, frequency of care recipient behavior problems; caregiver self-efficacy, sense of competence, distress; acceptability, feasibility, and perceived utility of system.  Use of RAM not significantly associated with changes in caregiver outcomes. | Tests if and how remote activity monitoring improves caregiver outcomes. |
|  | Mitchell et al., 2020 | Journal Article | Mixed-Methods Survey Study/ Interview Study (within RCT) | Persons with ADRD and their caregivers | **Remote activity, lifestyle, and health monitoring; sensor alert systems; emergency/assistance pendants/buttons** | N1=30 caregivers and care recipient s with ADRD randomized to intervention condition for survey  N2=7 semi-structured interviews | Acceptability and utility of RAM system (so significant correlation to demo variables, dementia severity, or caregiver distress/resources). | Assesses acceptability and utility of a remote activity monitoring system |
|  | Zmora et al., 2021 | Journal Article | Mixed-methods (Survey Study & Intervention Alert Data) (within RCT) | Persons with ADRD and their caregivers | **Remote activity, lifestyle, and health monitoring; sensor alert systems; emergency/assistance pendants/buttons** | N=36 caregivers of people living with dementia and their care recipient s | Caregiver self-efficacy, sense of competence, burden, role captivity, role overload  Perceived usefulness and ease of use | Describes patterns of remote activity monitoring system alerts and their association with perceived utility and acceptability of the system. |
| 21 | Giebel et al., 2021 | Journal Article | Qualitative Interview Study | Dementia caregivers | **Hiring Formal Care Services/Homes/ Professionals** | N=16 unpaid dementia caregivers (9/16 lived separate from people living with dementia) | Caregiver experiences accessing care and services during COVID-19 | Explores caregiver experiences accessing care and services during COVID-19 |
| 22 | Gillani & Arslan, 2021 | Journal Article | Systematic Review | Persons with Alzheimer’s disease | Remote activity, lifestyle, and health monitoring; sensor alert systems; localization & GPS tracking; reminder devices & memory aids; reminder apps | N/A | N/A | Summarizes intelligent sensing systems for the management of Alzheimer’s disease |
| 23 | Goodman-Casanova et al., 2019 | Journal Article (Protocol Paper) | RCT (Quantitative) | Patients with cognitive impairment and their caregivers | **Reminder devices & memory aids; calendars/scheduling; video communication; physiological monitoring** | N=240 dyads of people with cognitive impairments and their caregivers (anticipated) | Care recipient/caregiver QoL, adherence and compliance to prescribed medication, functional status, service utilization, and caregiver burden. Usability evaluation of intervention.  (Anticipated) | Describes a study protocol for a 12-month RCT using a TV-based platform service to support patients with mild cognitive impairment or dementia |
| 24 | Gunn et al., 2021 | Journal Article | Qualitative Interview Study | Long-distance caregivers of older adults | **Hiring Formal Care Services/Homes/ Professionals** | N=13 adult long-distance caregivers for older adults in Australia | Factors influencing choice of aged care provider, experiences with aged care provider. | Investigates factors that influence how long-distance caregivers select aged care providers |
| 25 | Koerin & Harrigan, 2003 | Journal Article | Quantitative Survey Study (Secondary Analysis) | Long-distance caregivers of older adults | Hiring Formal Care Services/Homes/ Professionals; | N=109 long-distance caregivers (1+ hour away from care recipient ) | N/A | Reports a secondary analysis of a 1997 survey; focused on long-distance caregivers |
| 26 | Lee & Chuang, 2021 | Conference Proceeding | Quantitative Proof-of-concept | People living with dementia | **Remote activity, lifestyle, and health monitoring; sensor alert systems; fall detectors; localization & GPS tracking** | N=25 professional caregivers and N=15 family caregivers | Caregiver satisfaction with use of the system (93% for family caregivers), assessment of usefulness of device, fall detection accuracy (94%). | Presents a remote disease care service platform with IoT devices to monitor location of older adults and alert to potential hazards. |
| 27 | Lewis, 2008 | Article (not peer-reviewed) | Case Studies (2) | Long-distance caregivers | Hiring Formal Care Services/Homes/ Professionals | N=2 Aging/ Alzheimer’s researchers who serve as long-distance caregivers for their family. | N/A | Presents two case studies of Aging/Alzheimer’s researchers who serve as long-distance caregivers themselves; provides tips for long-distance caregivers |
| 28 | Liu et al., 2017 | Journal Article | Mixed-methods Questionnaire/Focus Group Study | People living with dementia and their caregivers | **Localization & GPS tracking; geofencing** | N=45 dyads of people living with dementia and their caregivers (only 29 people living with dementia were able to complete exit questionnaire)  Focus groups N=15 caregivers and 9 stakeholders (police, care management, social workers, etc.) | Device use behavior; acceptance of technology (UTAUT factors); experience using tech; device usefulness (opinion) | Uses mixed-methods approach to determine acceptance of wearable GPS tech for community-based individuals with dementia for wandering |
| 29 | Mahoney, 2011 | Journal Article | Meta-synthesis | Older adults and their caregivers | Remote activity, lifestyle, health monitoring; sensor alert systems; | Dementia telecaregiving-specific studies  N=27 dyads | Factors influencing the adoption of technology for remote monitoring of older adults’ daily activities | Identifies key variables the influence the adoption of remote monitoring technologies in “real world” homes |
| 30 | Marling, 2007 | Book Chapter | Review | Older adults | Remote activity, lifestyle, and health monitoring; sensor alert systems; video monitoring; home security; controllers & switches; reminder devices & memory aids; calendars/ scheduling; | N/A | N/A | Outlines intelligent systems for enhancing older adult quality of life |
| 31 | Megges et al., 2017 | Journal Article | Quantitative Prototype pilot study | People living with dementia and their caregivers | **Localization & GPS tracking; geofencing** | N=17 dyads of people living with dementia and their caregivers | Device usability, ratings of device functions and features; caregiver burden, perceived self-efficacy; frequency of use, willingness to purchase prototype | Testing GPS localization prototype app for usability with people living with dementia. |
| 32 | Milligan et al., 2011 | Journal Article | Qualitative Ethnographic Study/ Deliberative Panel | Older adults | Reminder devices & memory aids; automated pill boxes; emergency/assistance pendants/buttons; remote activity, lifestyle, and health monitoring; sensor alert systems; home security; controllers & switches | 2 projects:  Two-day event that brought together N=60 providers. 2 UK-based focus groups with 6-8 frail older adults and their caregivers  8 deliberative panels with older adults (8-10 participants in each), ethnographic interviews in homes of 8 older adult telecare users, interviews with 10 key actors from social care services and telecare design companies. | N/A  (theoretical discussion) | Considers how telecare developments contribute to reshaping the care of older adults |
| 33 | Milne et al., 2014 | Journal Article | Mixed-Methods Feasibility Study | People living with dementia and their caregivers | **Localization & GPS tracking; geofencing** | N=12 dyads of people living with dementia and their caregivers (12 caregivers and 8 people living with dementia in first interview; 8 caregivers and 4 people living with dementia in followups)  N=15 professional stakeholders for focus groups | Perceived utility & challenges of GPS; caregiver QoL, caregiver stress; time spent searching for care recipients; number of wandering episodes; admission to/days in nursing/residential homes or long-term care; hospital visits/admissions/out of hospital doctor contacts; economic evaluation (if the intervention is cost effective when compared to normal care) | Feasibility trial of a GPS technology to assess feasibility of an RCT |
| 34 | Nishiura et al., 2021 | Journal Article | Mixed-methods Cross-over RCT | Older adults with and without dementia | **Reminder devices & memory aids; calendars/scheduling** | N=27 older adults (n=20 with some form of dementia) (n=23 included in analysis due to attrition) | Daily activities related to healthcare, cognitive function | Tests the use of an electric calendar for older adults with and without dementia to help with dementia symptoms (disorientation, memory, etc.) |
| 35 | Øderud et al., 2015 | Journal Article | Mixed-methods Technology Evaluation | People living with dementia | **Localization & GPS tracking** | N=208 people living with dementia (50% had informal caregivers using GPS to locate people living with dementia) | Experiences of caregivers and care recipients using intervention | Evaluating the experience of using GPS intervention for people living with dementia |
| 36 | Olsson et al., 2013 | Journal Article | Qualitative Intervention Pilot Study | People living with dementia and their spouses | **Localization & GPS tracking; geofencing** | N=5 dyads of people living with dementia and their spouses | Experiences using intervention | Ethnographic and interview study exploring the use of localization intervention for people living with dementia and their spouses |
|  | Olsson et al., 2016 | Journal Article | Qualitative Exploratory Pilot (Interview Study) | People living with dementia | **Localization & GPS tracking; geofencing** | N=11 people with mild dementia | Perceptions/opinions of people living with dementia on the GPS intervention demonstrated to them | Repeated interview study with people with mild dementia to get their perceptions/opinions on the use of a GPS tracker intervention |
| 37 | Pappadà et al., 2021 | Journal Article | Systematic Review | People living with dementia and their caregivers | Reminder devices & memory aids; task prompters; automated pill boxes; facial recognition reminders; calendars/scheduling; video, messaging, telephone communication; telepresence robots; localization & GPS tracking; remote activity, lifestyle, and health monitoring; sensor alert systems; video monitoring | N/A | N/A | Reviews technology to support people living with dementia and their caregivers |
| 38 | Pot et al., 2012 | Journal Article | Quantitative Pilot Study | People living with dementia and their caregivers | **Localization & GPS tracking; telephone communication** | N=28 dyads of people living with dementia and their caregivers | Caregiver impression of device; device acceptability (caregiver & care recipient ); caregiver & care recipient device usability; caregiver role overload; care recipient self-reported worry; caregiver self-reported worry | Pilot study of a GPS intervention with a calling option for people living with dementia and their caregivers |
| 39 | Rajasekaran et al., 2011 | Conference Proceeding | Quantitative Pilot Study | Older adults with cognitive impairment | **Remote activity, lifestyle, and health monitoring; physiological monitoring** | N=6 healthy volunteers | System reliability | Outlines a device to monitor and detect negative behavioral and psychology symptoms in persons with cognitive impairment |
| 40 | Riehle et al., 2011 | Conference Proceeding | Quantitative Technology Presentation | Persons with cognitive impairment | **Localization & GPS tracking; transportation assistance** | N/A | Algorithm performance | Presents a software to help people with cognitive impairment use public transportation |
| 41 | Rowe et al., 2009 | Journal Article | Quantitative Pilot Study | People living with dementia and their caregivers | **Remote activity, lifestyle, and health monitoring; sensor alert systems** | N=53 dyads of people living with dementia and caregivers (n=26 in experimental, n=27 in control)  Completed study: n=16 experimental, n=17 in control | System reliability; caregiver satisfaction with system; care recipient nighttime injuries; care recipient exits from home | Pilot study of a nighttime monitoring system for informal caregivers of people living with dementia |
| 42 | Smith, 2006 | Journal Article | Case Study | Long-distance caregivers of older adults | N/A | N/A | N/A | Examines the impact of long-distance caregiving on family members and the care recipient and how long-distance caregivers can be better supported |
| 43 | Stavropoulos et al., 2020 | Journal Article | Systematic Review | Older adults and their caregivers | Emergency/assistance pendants/buttons; localization & GPS tracking; remote activity, lifestyle, and health monitoring; sensor alert systems; fall detectors; physiological monitoring | N/A | N/A | Reviews IoT wearables and devices to support older adults and their caregivers |
| 44 | Tiersen et al., 2021 | Journal Article | Interview Study/ Workshop/ Focus Group Study (Qualitative) | People living with dementia and their caregivers and other stakeholders | N/A | 9 sub-studies:  Semi-structured interviews with n=9 people living with dementia and their caregivers and n=10 academic and clinical staff  Workshops with 35 people living with dementia and their caregivers, 12 health and social care clinicians  Workshops with 24 occupational therapistss, NHS pathway directors and researchers  2 focus groups with managers of health care services (n=8)  Semi-structured interviews with 10 caregivers and 2 people living with dementia | Care recipient personal development & need assessment, opportunities for new care technologies and strategies | Investigates the needs of people living with dementia, their caregivers, and other stakeholders for smart home systems |
| 45 | Vincent et al., 2002 | Journal Article | Mixed-Methods Technology Trial | Persons with moderate cognitive problems or significant functional limitations and their caregivers | **Reminder devices & memory aids; calendars/scheduling; emergency/assistance pendants/buttons; remote activity lifestyle, and health monitoring; home security; automatic/ remote door locks; lights & smart plugs, outdoor intercoms** | N=5 users with moderate cognitive problems or significant functional limitations and their caregivers | Caregiver burden (lessened for psychological aspects but not physical ones), participant satisfaction, intervention effectiveness (works or not).  Users generally satisfied. | Examines the application of new environmental control systems in the homes of users and caregivers |
| 46 | Wang et al., 2021 | Journal Article | Systematic Review | Long-distance caregivers of community-dwelling older adults | Remote activity, lifestyle, and health monitoring; fall detectors | N/A | Older adult attitudes towards and intention to use long-distance caregiving technology. | Reviews factors influencing attitudes towards and intention to use long-distance caregiving technologies for older adults |
| 47 | Watari et al., 2006 | Journal Article | Mixed-Methods Survey Study | Long-distance caregivers of people living with dementia in Los Angeles County | **Organization developed or led long-distance caregiver programs** | n=90 long-distance caregivers of people living with dementia and n=187 local caregivers | Available services used, satisfaction with services | Compares demographics and use of Alzheimer’s Association of Los Angeles services between 90 long-distance caregivers and 187 local caregivers |
| 48 | Zwierenberg et al., 2018 | Journal Article | Qualitative Interview Study/ Technology Field Study | People living with dementia and their caregivers and care managers | **Remote activity, lifestyle, and health monitoring; sensor alert systems** | N=50 informal caregivers of people living with dementia and N=13 care managers (First interview)  N=41 caregivers and N=7 case managers for second interview | User experiences (impact of system on care, impressions of usefulness, etc., benefits, concerns etc.) | Investigates needs, benefits, and concerns relating to lifestyle monitoring systems |
| **Non-Study Sources** | | | | | | | | |
| **Source** | **Citation** | **Source Type** | **Study Design** | **Target Population** | **Intervention(s):** Discussed  **Tested** | **Sample Size** | **Tested Outcomes** | **Brief Description** |
| 1 | Alzheimer’s Association, 2013 | Journal Article | Statistical Resource  (Non-study) | People living with dementia and their caregivers | N/A | N/A | N/A | US data related to Alzheimer’s disease; focuses specifically on long-distance caregivers |
| 2 | Faieta et al., 2021 | Journal Article | Information/ Education Page  (Non-study) | Older adults and their caregivers | Reminder devices & memory aids; reminder apps; alarms; video, messaging, telephone communication; remote activity, lifestyle, and health monitoring; physiological monitoring; home security; cameras & video doorbells; automatic/remote locks; controllers & switches; lights & smart plugs | N/A | N/A | Briefly overviews technologies for remote caregiving |
| 3 | Lv et al., 2020 | Journal Article | Technology Presentation  (non-study) | People living with dementia and their caregivers | **Telerobotics** | N/A | N/A | Presents a dual-arm telerobotic system for caregivers to remotely assist people living with dementia |
| 4 | Wolff et al., 2021 | Journal Article | Panel Summary (non-study) | People living with dementia | Reminder devices & memory aids; video, messaging, and telephone communication; localization & GPS tracking; remote activity, lifestyle, and health monitoring; video monitoring | N=3 researchers (one of whom presented on technology for dementia care) | N/A | Reviews 3 areas of emerging science in dementia care including one regarding technology to aid in dementia caregiving |
| 5 | Zagaria, 2012 | Journal Article | Tip Sheet/ Educational Resource  (non-study) | Long-distance caregivers | Hiring Formal Care Services/Homes/ Professionals; video, messaging, and telephone communication; fall prevention checklist | N/A | N/A | Article advising nurse practitioners how they can counsel long-distance caregivers |

## Data Extraction Tool

See supplementary materials for Excel file.

## PRISMA Checklist

| **SECTION** | **ITEM** | **PRISMA-ScR CHECKLIST ITEM** | **REPORTED ON PAGE #** |
| --- | --- | --- | --- |
| **TITLE** | | | |
| Title | 1 | Identify the report as a scoping review. | 1 (mapping) |
| **ABSTRACT** | | | |
| Structured summary | 2 | Provide a structured summary that includes (as applicable): background, objectives, eligibility criteria, sources of evidence, charting methods, results, and conclusions that relate to the review questions and objectives. | 2 |
| **INTRODUCTION** | | | |
| Rationale | 3 | Describe the rationale for the review in the context of what is already known. Explain why the review questions/objectives lend themselves to a scoping review approach. | 3-4 |
| Objectives | 4 | Provide an explicit statement of the questions and objectives being addressed with reference to their key elements (e.g., population or participants, concepts, and context) or other relevant key elements used to conceptualize the review questions and/or objectives. | 3 |
| **METHODS** | | | |
| Protocol and registration | 5 | Indicate whether a review protocol exists; state if and where it can be accessed (e.g., a Web address); and if available, provide registration information, including the registration number. | N/A |
| Eligibility criteria | 6 | Specify characteristics of the sources of evidence used as eligibility criteria (e.g., years considered, language, and publication status), and provide a rationale. | 4 |
| Information sources* | 7 | Describe all information sources in the search (e.g., databases with dates of coverage and contact with authors to identify additional sources), as well as the date the most recent search was executed. | 4 |
| Search | 8 | Present the full electronic search strategy for at least 1 database, including any limits used, such that it could be repeated. | 4 |
| Selection of sources of evidence† | 9 | State the process for selecting sources of evidence (i.e., screening and eligibility) included in the scoping review. | 4 |
| Data charting process‡ | 10 | Describe the methods of charting data from the included sources of evidence (e.g., calibrated forms or forms that have been tested by the team before their use, and whether data charting was done independently or in duplicate) and any processes for obtaining and confirming data from investigators. | 4 |
| Data items | 11 | List and define all variables for which data were sought and any assumptions and simplifications made. | N/A |
| Critical appraisal of individual sources of evidence§ | 12 | If done, provide a rationale for conducting a critical appraisal of included sources of evidence; describe the methods used and how this information was used in any data synthesis (if appropriate). | N/A |
| Synthesis of results | 13 | Describe the methods of handling and summarizing the data that were charted. | 4 |
| **RESULTS** | | | |
| Selection of sources of evidence | 14 | Give numbers of sources of evidence screened, assessed for eligibility, and included in the review, with reasons for exclusions at each stage, ideally using a flow diagram. | 4, 27 |
| Characteristics of sources of evidence | 15 | For each source of evidence, present characteristics for which data were charted and provide the citations. | 19-25 |
| Critical appraisal within sources of evidence | 16 | If done, present data on critical appraisal of included sources of evidence (see item 12). | N/A |
| Results of individual sources of evidence | 17 | For each included source of evidence, present the relevant data that were charted that relate to the review questions and objectives. | 19-25 |
| Synthesis of results | 18 | Summarize and/or present the charting results as they relate to the review questions and objectives. | 4-10, 28 |
| **DISCUSSION** | | | |
| Summary of evidence | 19 | Summarize the main results (including an overview of concepts, themes, and types of evidence available), link to the review questions and objectives, and consider the relevance to key groups. | 10-12 |
| Limitations | 20 | Discuss the limitations of the scoping review process. | 12 |
| Conclusions | 21 | Provide a general interpretation of the results with respect to the review questions and objectives, as well as potential implications and/or next steps. | 10-12 |
| **FUNDING** | | | |
| Funding | 22 | Describe sources of funding for the included sources of evidence, as well as sources of funding for the scoping review. Describe the role of the funders of the scoping review. | 12-13 |

From: Tricco AC, Lillie E, Zarin W, O'Brien KK, Colquhoun H, Levac D, et al. PRISMA Extension for Scoping Reviews (PRISMAScR): Checklist and Explanation. Ann Intern Med. 2018;169:467–473. doi: 10.7326/M18-0850.
